# Association between arterial stiffness and left ventricular diastolic function: A large population-based cross-sectional study

**DOI:** 10.3389/fcvm.2022.1001248

**Published:** 2022-10-13

**Authors:** Minkwan Kim, Hack-Lyoung Kim, Woo-Hyun Lim, Jae-Bin Seo, Sang-Hyun Kim, Myung-A Kim, Joo-Hee Zo

**Affiliations:** ^1^Division of Cardiology, Department of Internal Medicine, Yongin Severance Hospital, Yonsei University College of Medicine, Yongin-si, South Korea; ^2^Division of Cardiology, Department of Internal Medicine, Boramae Medical Center, Seoul National University College of Medicine, Seoul, South Korea

**Keywords:** pulse wave velocity, diastolic function, ventricular-vascular coupling, arterial stiffness, heart failure with a preserved ejection fraction

## Abstract

**Background:**

The association between arterial stiffness and left ventricular (LV) diastolic function has been demonstrated in several studies, but the samples size in those studies was small. This study aims to verify this issue in a large number of study subjects.

**Methods:**

A total of 7,013 consecutive participants (mean age 60.6 years and 43.3% female) who underwent both baPWV and transthoracic echocardiography were retrospectively analyzed. Subjects with significant cardiac structural abnormalities were excluded.

**Results:**

There were significant correlations of baPWV with septal e′ velocity (*r* = – 0.408; *P* < 0.001), septal E/e′ (*r* = 0.349; *P* < 0.001), left atrial volume index (LAVI) (*r* = 0.122; *P* < 0.001) and maximal velocity of tricuspid valve regurgitation (TR Vmax) (*r* = 0.322; *P* < 0.001). The baPWV values increased proportionally with an increase in the number of LV diastolic indices meeting LV diastolic dysfunction criteria (*P*-for-trend < 0.001). In multivariable analyses with adjustment for confounding effects of various clinical covariates, higher baPWV was independently associated with septal e′ < 7 (odds ratio [OR], 1.30; 95% confidence interval [CI] 1.20–1.60; *P* < 0.001), septal E/e′ ≥ 15 (OR, 1.46; 95% CI, 1.21–1.78; *P* < 0.001), and TR Vmax > 2.8 m/s (OR, 1.60; 95% CI, 1.23–2.09; *P* < 0.001) but not with LAVI ≥ 34 mL/m^2^ (OR, 0.89; 95% CI, 0.76–1.03; *P* = 0.123).

**Conclusions:**

Increased arterial stiffness, as measured by baPWV, was associated with abnormal diastolic function parameters in a large number of study participants, providing strong evidence to the existing data about ventricular-vascular coupling.

## Introduction

As the elderly population gradually increases, the prevalence of heart failure (HF) is increasing worldwide (1). Because the treatment strategies are very different, HF is divided into two major groups according to left ventricular ejection fraction (LVEF): HF with preserved ejection fraction (HFpEF) and HF with reduced ejection fraction (HFrEF) (2). The pathophysiology of the occurrence and deterioration of HFrEF is relatively well known; based on this, many effective drugs have been developed to improve the long-term prognosis of HFrEF patients. However, the pathogenesis of HFpEF has not yet been well elucidated, and drugs that improve the long-term prognosis of patients with HFpEF are limited.

It has been suggested that abnormal ventricular-vascular coupling (VVC) may be involved in the development of HFpEF (3, 4). Arterial stiffening increases afterload of left ventricle (LV) leading to LV hypertrophy. In addition, decreases in coronary perfusion by reduced diastolic pressure in stiffened arteries aggravate subendocardial ischemia. Shared common risk factors between arterial stiffening and LV diastolic dysfunction is another suggested mechanism for VVC (5). On this theoretical background, many studies have shown a correlation between arterial stiffness and LV diastolic function (6–8). However, the study sample size was relatively small in those studies. Most of the subjects analyzed in the studies ranged from tens to hundreds at most. By analyzing a large number of study subjects, errors can be reduced and clearer results can be drawn (9). We intended to present stronger evidence for the association between arterial stiffness and LV diastolic function by analyzing a large number of study subjects. Accordingly, this study was performed to investigate the association between the measures of arterial stiffness and LV diastolic function in ~7,000 subjects.

## Methods

### Study population

Between October 2008 and December 2019, among 11,767 candidates available baPWV data, we retrospectively reviewed the medical records of 8,348 participants who underwent both baPWV and transthoracic echocardiography within 7 days at a single cardiovascular center. The attending physician measured baPWV for cardiovascular risk assessment. We excluded the following conditions to increase the reliability of baPWV or diastolic function parameters (*n* = 1,335): (1) ankle-brachial index < 0.9, (2) LV ejection fraction < 50%, (3) supraventricular or ventricular arrhythmias, (4) valvular heart disease more than a mild degree, (5) significant pericardial effusion, and (6) congenital heart disease. Finally, 7,013 participants were enrolled in the present study (Supplementary Figure 1). This study was conducted in accordance with the declaration of Helsinki, revised in 2013. The study protocol was approved by the Institutional Review Board (IRB) of Boramae Medical Center (Seoul, Republic of Korea) (IRB number: 20-2022-72), and informed consent was waived due to the study's retrospective nature.

### Data collection

The following clinical data were collected by reviewing medical records: (1) body weight and height; (2) systolic and diastolic blood pressure; (3) heart rate; (4) history of hypertension, diabetes mellitus, dyslipidemia, current smoking (cigarette smoking within 12 months), coronary artery disease (myocardial infarction and coronary revascularization) and stroke. Body mass index was calculated as weight (kg)/ square height (m^2^), and body mass index ≥ 25 kg/m^2^ was considered obese (10). The following information on blood test results was also obtained: white blood cell count, hemoglobin, fasting glucose, glycated hemoglobin, estimated glomerular filtration rate (GFR) by Modification of Diet in Renal Disease (MDRD) equation, lipid profiles of total cholesterol, low- and high-density lipoprotein, triglyceride, and C-reactive protein. We also reviewed concomitant vasoactive medications, including calcium channel blockers, beta-blockers, renin-angiotensin system blockers and statins.

### Measurement of baPWV

On the day of the measurement of baPWV, participants were forbidden from smoking, alcohol, and caffeine-containing beverages such as coffee. Medications were not interrupted if administrated regularly and continued to be taken. Before the examination, participants were asked to rest in bed for about 5 mins. The baPWV was measured using a commercially available device (VP-1000 analyzer: Colins, Komaki, Japan). The brachial and tibial arteries were the points obtaining the pressure waveforms using plethysmographic and oscillometric pressure sensors with occlusion/sensing cuffs. The time intervals between pressure waveforms of the brachial and tibial arteries (pulse transit time) were measured, and baPWV was calculated automatically based on the patient's height. We used the mean of right and left baPWV values for the analysis of this study. The baPWV measurement was performed by a single experienced operator. The coefficient of variation in baPWV measurement for intraobserver variability was 5.1% in our laboratory (11).

### Transthoracic echocardiography

Transthoracic echocardiographic examination was performed with commercially available equipment (EPIQ 7 and EPIQ CVx; Philips Ultrasound, Inc., Bothell, WA, USA or Vivid E9 and Vivid E95; GE Healthcare, Horten, Norway). LV dimension and wall thickness were measured using M-mode technique. LV ejection fraction was measured using biplane Simpson's method. Relative wall thickness was calculated as 2 × LV posterior wall thickness/LV end-diastolic dimension. LV mass was calculated using the Cube formula, and indexed by body surface area (= LV mass index) (12). For the evaluation of diastolic function, we focused on 4 diastolic indices [septal e′ velocity, E/e′, left atrial volume index (LAVI), and maximal velocity of tricuspid regurgitation (TR Vmax)] according to the current guidelines (13). Mitral inflow velocities in both early and atrial phases were obtained using a pulsed wave Doppler at the mitral leaflet tip in an apical 4-chamber view. Tissue Doppler velocities were obtained at the medial mitral annulus. The LAVI was calculated using the biplane method and indexed by body surface area. TR Vmax was measured in a modified 4-chamber view. The criteria for each parameter suggesting left ventricular diastolic dysfunction are as follows: septal e′ velocity < 7 cm/s, septal E/e′ ≥ 15, LAVI ≥ 34 ml/m^2^ and TR Vmax > 2.8 m/s (13). Diastolic function was graded based on the recommendations of current guidelines: >50% positive among four variables means the patient had diastolic dysfunction (II or III), and indeterminate was defined as two of four variables were satisfied with previously noted abnormal values; < 50% means normal diastolic function; Grade I diastolic function was defined as participants with the myocardial disease, regional wall motion abnormality, history of hypertension and enlarged left atrial or increased left ventricular wall thickness, but not meeting the criteria more than grade II diastolic dysfunction (13). The interobserver coefficients of variation for e′ and E/e′ were 6.8 and 7.0%, respectively, in our laboratory (14).

### Statistical analysis

Categorical variables are presented as numbers (percentages), and continuous variables as mean ± standard deviation. Comparisons of categorical variables were analyzed using the Chi-squared test, and Student's *t*-test for continuous variables. Linear correlation between baPWV and diastolic parameters were analyzed using Pearson's correlation analysis, and the results were also demonstrated using scatter plots. The trend of changes in baPWV among the groups according to the number of abnormal diastolic parameters and diastolic dysfunction grades were analyzed by the Cochran–Armitage test. We also analyzed the trend toward an increase of baPWV across the diastolic function grade in subpopulation after excluding participants who had indeterminate diastolic function based on the 2016 diastolic function guideline. Receiver operating characteristics curve analysis was performed to determine the optimal cutoff value of baPWV for diastolic function parameters based on the Youden index (15). The missForest algorithm was used to perform missing data imputation. We used all the available variables to perform the imputation (16). Multivariable analyses (binary logistic and linear regression analyses) were performed to investigate the independent association between baPWV and diastolic parameters with adjustment for confounding effects of several important clinical covariates. We performed random forest analysis to determine the relative importance of baPWV in affecting each diastolic function parameter among demographic, clinical, and laboratory variables (17). In briefly, random forest analysis is one of supervised machine learning method and classification algorithm that consists of a large number of individual decision trees that perform as an ensemble: each individual tree in the random forest spits out a class prediction and the class with the most votes becomes our model's prediction (17). Original cohort was divided into the derivation (65%) and the validation (35%) cohort, to test the optimized model created from the derivation cohort with the area under curve in the receiver operating characteristic analysis. The relative importance of each variable was analyzed by the classification error for the random forest trees and the error after permuting the predictor variables (17). A two-tailed *P* < 0.05 was considered significant. All statistical analyses were performed using R version 4.0.6 software (R Development Core Team, Vienna, Austria).

## Results

### Clinical characteristics as well as echocardiographic and baPWV findings

The baseline clinical characteristics of study participants are shown in Table 1. The mean age of study subjects was 60.6 ± 12.2 years, and 43.3% were female. The prevalence of hypertension, diabetes mellitus, dyslipidemia, current smoking and obesity were 47.5, 21.7, 49.5, 18.7, and 45.1%, respectively. Coronary artery disease was identified in 1,953 subjects (27.8%), and stroke in 36 subjects (0.5%). Most of the major blood test findings were within the normal range. The proportion of subjects taking calcium channel blockers, beta-blockers, renin-angiotensin system blockers and statins were 13.8, 25.3, 32.0, and 44.3%, respectively.

**Table 1:** Clinical characteristics of study subjects.

**Characteristic**	**Value (*n* = 7,013)**
Age, years	60.6 ± 12.2
Female sex	3,035 (43.3)
Weight, kg	65.1 ± 11.5
Height, cm	161 ± 9
Body mass index, kg/m^2^	24.8 ± 3.3
Systolic blood pressure, mmHg	129 ± 18
Diastolic blood pressure, mmHg	77.2 ± 11.3
Heart rate, beats per minutes	69.3 ± 12.2
**Cardiovascular risk factors**	
Hypertension	3,331 (47.5)
Diabetes mellitus	1,519 (21.7)
Dyslipidemia	3,474 (49.5)
Current smoker	1,312 (18.7)
Obesity (body mass index ≥ 25 kg/m^2^)	3,150 (45.1)
Previous coronary artery disease	1,953 (27.8)
Previous stroke	36 (0.5)
**Laboratory findings**	
White blood cell count, μl	7,094 ± 3,201
Hemoglobin, g/dl	13.5 ± 1.8
Fasting glucose, mg/dl	118 ± 38
Glycated hemoglobin, %	6.2 ± 1.0
Estimated glomerular filtration rate, ml/min/1.73 m^2^	86.4 ± 25.3
Total cholesterol, mg/dl	168 ± 42
Low-density lipoprotein, mg/dl	100 ± 37
High-density lipoprotein, mg/dl	48.7 ± 13.1
Triglyceride, mg/dl	135 ± 103
C-reactive protein, mg/dl	1.3 ± 4.0
**Concomitant vasoactive medications**	
Calcium channel blockers	965 (13.8)
Beta-blockers	1,771 (25.3)
Renin-angiotensin system blockers	2,241 (32.0)
Statins	3,108 (44.3)

Numbers are presented as mean ± SD or n (%).

The results of transthoracic echocardiography and baPWV are presented in Table 2. The mean value of ejection fraction was 65.2%, within the normal range. The level of E over A was slightly lower, and deceleration time was delayed. The mean value of septal e′ velocity and E/e′ was 6.4 cm/s, and 10.9, respectively. Almost half of the participants had diastolic dysfunction (49.0%). The mean value of baPWV in the total population was 15.9 ± 3.3 m/s.

**Table 2:** Results of echocardiography and baPWV.

	**Value (*n* = 7,013)**
LV end-diastolic dimension, mm	48.4 ± 4.2
LV end-systolic dimension, mm	30.9 ± 4.4
LV ejection fraction, %	65.2 ± 6.2
Relative wall thickness	0.38 ± 0.05
LV mass index, g/m^2^	96.9 ± 25.4
E wave velocity, cm/s	65.3 ± 18.1
A wave velocity, cm/s	75.8 ± 19.9
E/A	0.9 ± 0.5
Deceleration time, ms	217.2 ± 54.5
Septal e′ velocity, cm/s	6.4 ± 2.2
Septal e′ velocity < 7 cm/s	4,016 (59.4)
Septal E/e′	10.9 ± 4.0
Septal E/e′≥ 15	880 (12.5)
LAVI, ml/m^2^	30.3 ± 10.5
LAVI ≥ 34 ml/m^2^	1,432 (20.4)
TR Vmax, m/s	2.3 ± 0.3
TR Vmax > 2.8 m/s	408 (5.8)
**Diastolic function grading**	
Normal	1,189 (17.0)
Indeterminate	2,387 (34.0)
I	2,894 (41.3)
II or III	543 (7.7)
baPWV, cm/s	1,594 ± 330

Numbers are presented as mean ± SD or n (%). baPWV, brachial-ankle pulse wave velocity; LV, left ventricle; LAVI, left atrial volume index; TR, tricuspid regurgitation.

### Association between baPWV and parameters of diastolic function

**Figure 1:**
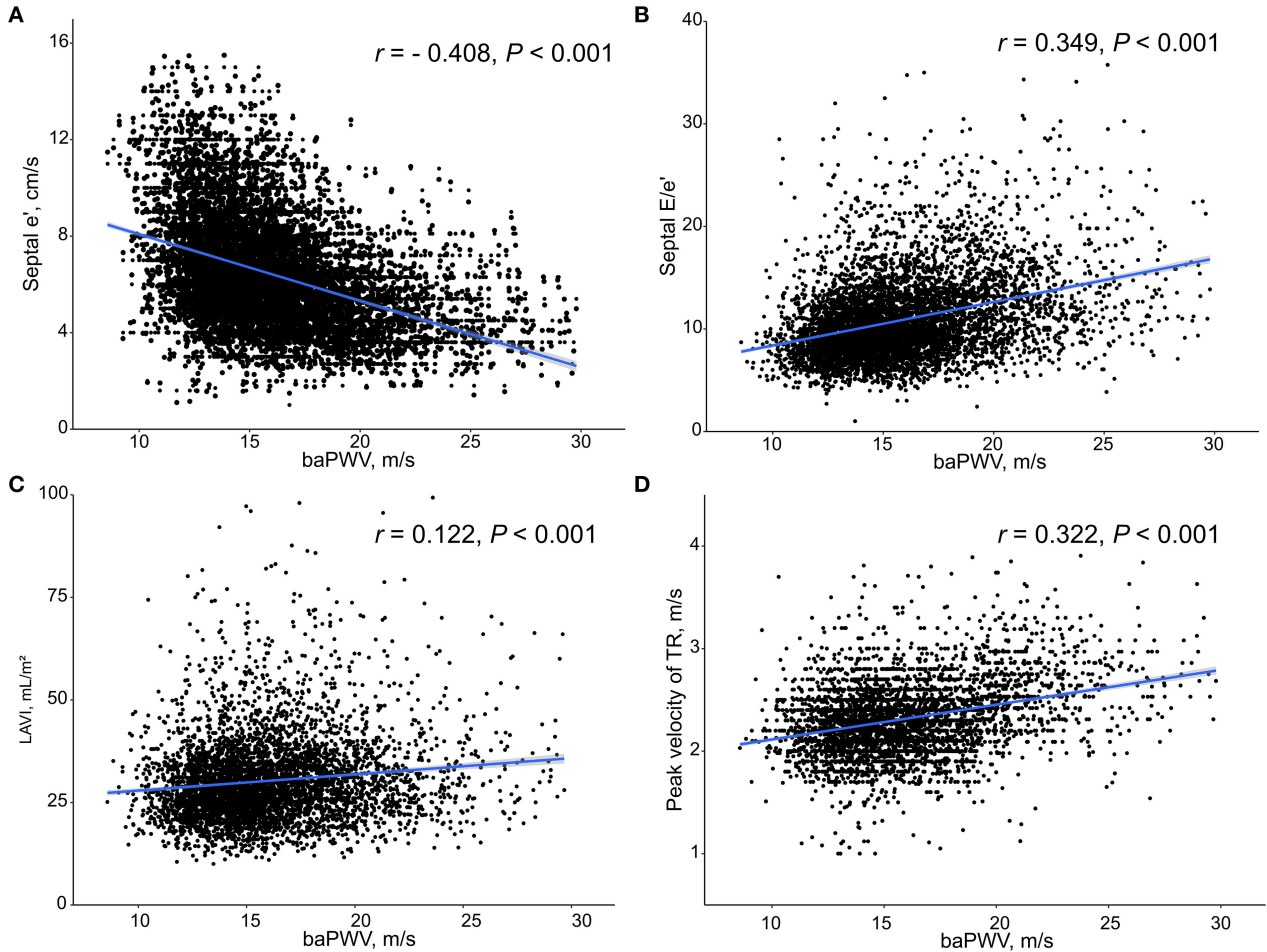
Scatter plots showing the associations between baPWV and diastolic function parameters including septal e′ velocity **(A)**, septal E/e′ **(B)**, LAVI **(C)** and peak velocity of TR **(D)**. baPWV, brachial-ankle pulse wave velocity; LAVI, Left atrial volume index; TR, tricuspid regurgitation.

**Figure 2:**
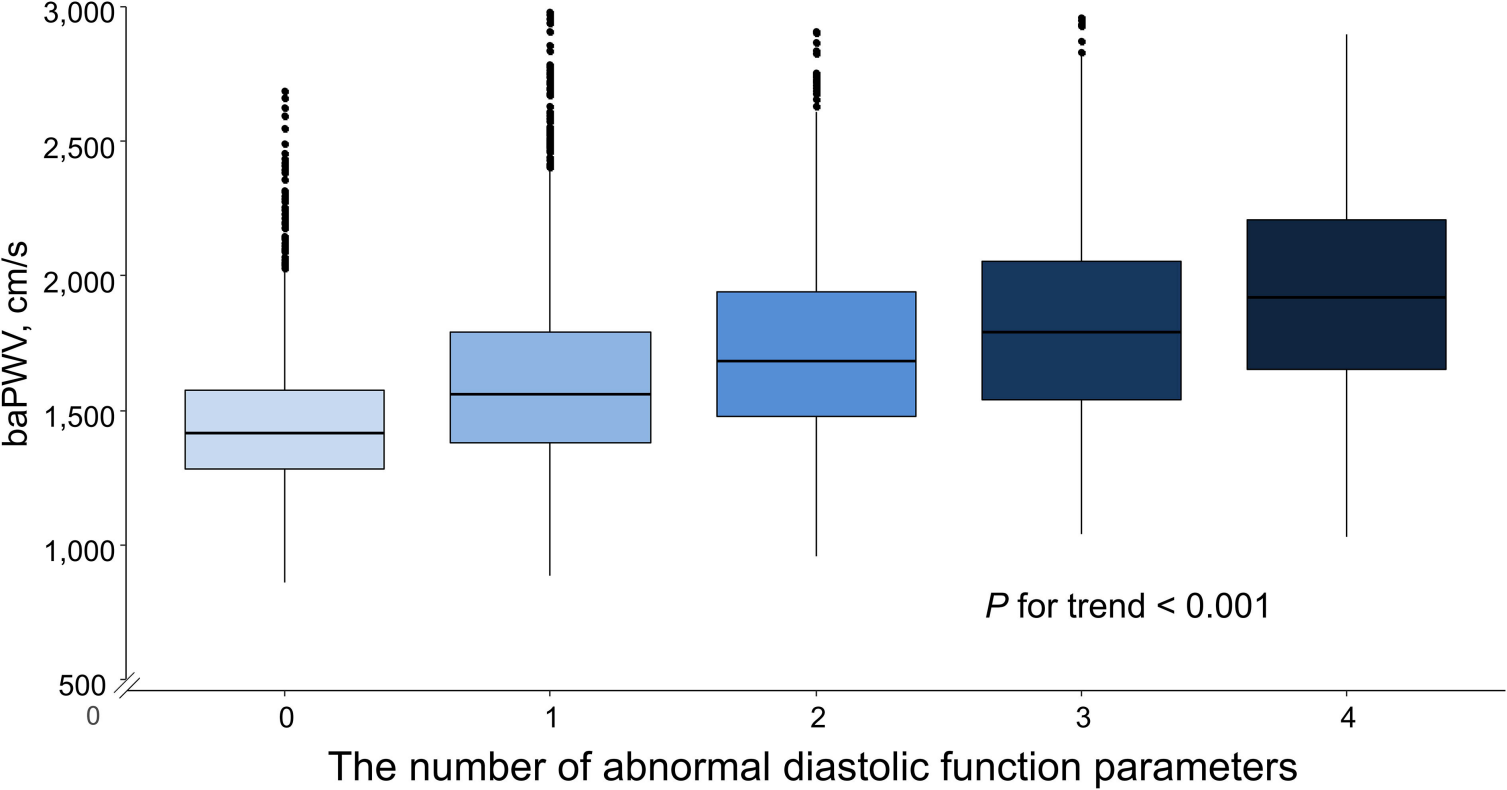
Box plot depicting the associations of the number of abnormal diastolic function parameters with baPWV. baPWV, brachial-ankle pulse wave velocity.

**Figure 3:**
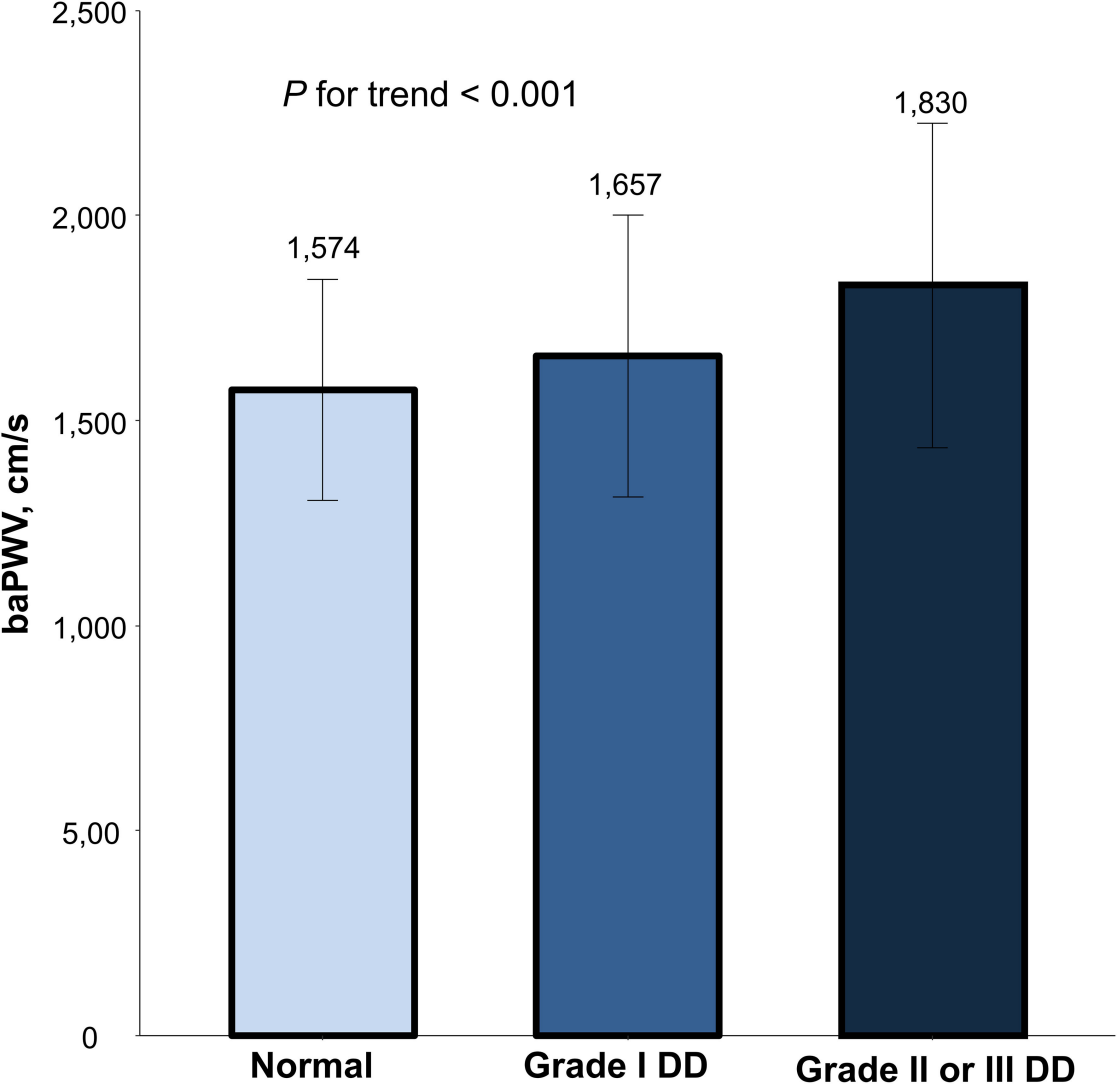
The mean and standard variation in baPWV according to diastolic dysfunction severity. Values are presented as mean and standard error. baPWV, brachial-ankle pulse wave velocity; DD, diastolic dysfunction.

Simple linear correlations between baPWV and diastolic function parameters are demonstrated in Figure 1: baPWV negatively correlated with septal e′ velocity (*r* = −0.408, *P* < 0.001), and positively correlated with septal E/e′ (*r* = 0.349, *P* < 0.001), LAVI (*r* = 0.122, *P* < 0.001) and TR Vmax (*r* = 0.322, *P* < 0.001). There was a positive association between baPWV and the number of diastolic parameters meeting the criteria for LV diastolic dysfunction: the higher the number of abnormal diastolic function parameters, the higher baPWV values (*P* for trend < 0.001) (Figure 2). There was a similar trend toward an increase of baPWV across the diastolic function grade in the subpopulation (*n* = 4,626) after excluding indeterminate diastolic function based on the 2016 diastolic function guideline (*P* for trend < 0.001) (Figure 3). The cutoff values of baPWV for septal e′ velocity < 7 cm/s, septal E/e′ ≥ 15, LAVI ≥ 34 ml/m^2^ and TR Vmax > 2.8 m/s were 16.1, 16.4, 16.3, and 17.7 m/s (sensitivity/specificity were 65.9/67.0% for septal e′; 52.0/77.8% for septal E/e′; 52.6/65.0% for LAVI; 58.6/77.0% for TR Vmax, respectively) (Supplementary Figure 2; Table 3). Using these cutoff values of baPWV, logistic regression analyses were performed. In univariable logistic regression analyses, increased baPWV was strongly correlated with all 4 abnormal diastolic function parameters (*P* < 0.05 for each). In multivariable logistic regression analyses, septal e′ < 7 cm/s [odd ratio (OR), 1.33; 95% confidence interval (CI): 1.15–1.54; *P* < 0.001], septal E/e′ ≥ 15 (OR, 1.38; 95% CI, 1.13–1.68; *P* < 0.001), TR Vmax > 2.8 m/s (OR, 2.07; 95% CI, 1.59–2.70; *P* < 0.001) were statistically significant to have an association with increased baPWV even after controlling for potential confounders including demographic, clinical, and laboratory covariates. However, there was no association between greater LAVI (≥ 34 ml/m^2^) and increased baPWV in this multivariable analysis (OR, 0.88; 95% CI, 0.75–1.03; *P* = 0.100) (Table 4). This trend also was seen in subgroup analysis according to the presence of cardiovascular risk factors or documented coronary artery disease (Supplementary Table 1).

**Table 3:** Binary logistic regression analysis showing the association between higher baPWV and abnormal diastolic parameters.

**Dependent variable**	**Unadjusted OR (95% CI)**	***P***	**Adjusted OR^†^ (95% CI)**	*P* *
Septal e′ < 7 cm/s	3.83 (3.48–4.34)	< 0.001	1.33 (1.15–1.54)	< 0.001
Septal E/e′≥ 15	3.83 (3.30–4.44)	< 0.001	1.38 (1.13–1.68)	< 0.001
LAVI ≥ 34 ml/m^2^	1.65 (1.47–1.86)	< 0.001	0.88 (0.75–1.03)	0.100
TR Vmax > 2.8 m/s	3.70 (2.99–4.58)	< 0.001	2.07 (1.59–2.70)	< 0.001

* P for baPWV ≥16.1 m/s in septal e′ < 7cm, baPWV ≥16.1 m/s in septal E/e′ ≥ 15, baPWV ≥16.3 m/s in LAVI ≥ 34 ml/m^2^, and baPWV ≥17.7 m/s in TR Vmax> 2.8 m/s, respectively.

^†^Four different multivariate analyzes were performed according to each dependent variable, and following clinical covariates were adjusted during the analysis: age, sex, body mass index, systolic blood pressure, heart rate, cardiovascular risk factors, hemoglobin, glomerular filtration rate, C-reactive protein, vasoactive medications, left ventricular ejection fraction, and left ventricular mass index. OR (95% CI) and P values are for in the association between baPWV and each diastolic parameter.

baPWV, brachial-ankle pulse wave velocity; OR, odds ratio; CI, confidence interval; LAVI, left atrial volume index; TR Vmax, maximal velocity of tricuspid regurgitation; LVMI, Left ventricular mass index.

**Table 4:** C-statistics, sensitivity and specificity of cutoff value for estimating abnormal diastolic function of baPWV.

	**C-statistic (95% CI)**	**Sensitivity**	**Specificity**
Septal e′ < 7 cm/s	0.71 (0.69–0.72)	65.9	67.0
Septal E/e′>15	0.71 (0.69–0.72)	52.0	77.8
LAVI >34 ml/m^2^	0.58 (0.56–0.59)	50.7	66.4
TR-Vmax > 2.8 m/s	0.71 (0.68–0.74)	58.6	77.0

Numbers of sensitivity and specificity are presented as percent (%). Cutoff value of baPWV was 16.1 m/s in septal e′ < 7 cm, 16.1 m/s in septal E/e′ ≥ 15, 16.3 m/s in LAVI ≥34 ml/m^2^, and 17.7 m/s in TR Vmax > 2.8 m/s, respectively.

### Relative importance of baPWV on diastolic dysfunction in comparison with other clinical variables

We performed random forest analysis to compare the relative importance of baPWV on each abnormal diastolic function parameter in relation to 28 other cardiovascular risk factors, including demographic, clinical, and laboratory covariates. The best predictive predicting model created from the derivation cohort was built on a model of tree 2000 in both septal E/e′ and septal e′ with an area under curve of 0.77 (95% CI, 0.75–0.79), and 0.78 (95% CI, 0.77–0.79), respectively, when assessed on the validation cohort.

**Figure 4:**
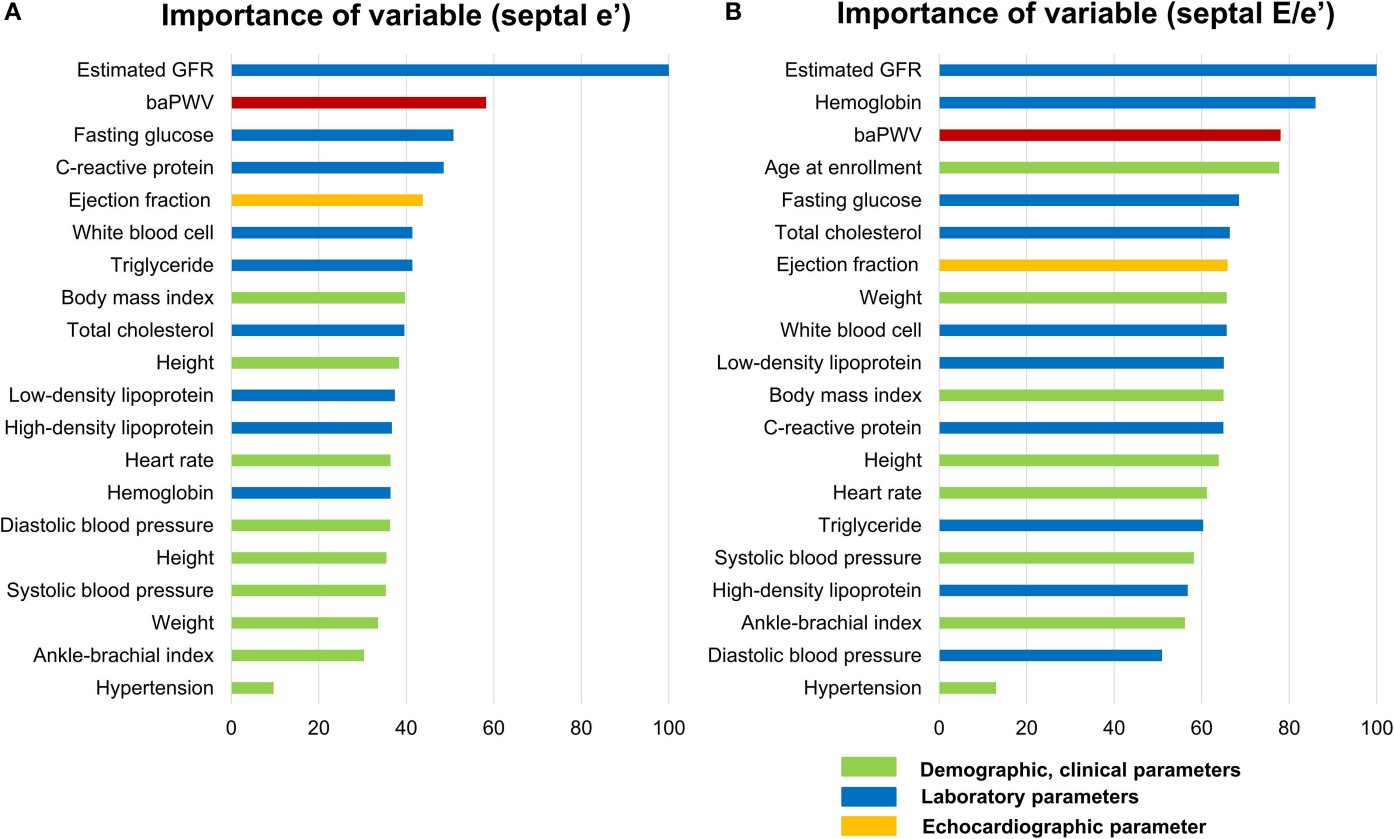
Relative importance of baPWV in predicting the abnormal diastolic function parameters septal E/e′**(A)** and septal e′ velocity **(B)** as analyzed in random forest analysis. Among the 29 variables, only the top 20 important variables are depicted. As the importance of the highest variable (estimated GFR in septal E/e′ and age in septal e′ velocity) was set as 100 and the relative importance of other variables was compared. baPWV, brachial-ankle pulse wave velocity; GFR, glomerular filtration rate.

Along with estimated GFR and hemoglobin, baPWV showed higher relative importance in affecting elevated septal E/e′ among cardiovascular risk factors (Figure 4A). Also, baPWV was the high-ranked importance of variable after age and estimated GFR among 29 covariates in affecting decreased septal e′ (Figure 4B). In both models, baPWV had higher importance in predicting abnormal diastolic function than systolic blood pressure, fasting glucose, or body mass index. More specifically, presuming the significance of the highest variable (estimated GFR in septal E/e′ and age in septal e′) to be 100, the relative importance of baPWV in diastolic dysfunction was 73.7 and 50.5, respectively. Systolic blood pressure, one of the most affecting parameters in both baPWV and diastolic function, had 55.2 and 34.6 in relative importance.

## Discussion

The present study demonstrated that arterial stiffness as measured by baPWV was significantly associated with diastolic function parameters in a large study population. Among 4 major parameters based on the 2016 diastolic function guideline, baPWV was independently related to septal e′, septal E/e′, and TR Vmax even after adjustment for demographic, clinical, and laboratory covariates. Also, baPWV showed higher importance in affecting septal e′ velocity and septal E/e′ among cardiovascular risk factors and clinical parameters. The association between increased arterial stiffness and LV diastolic dysfunction has been shown in previous studies, but only a small number of patients have been analyzed in those studies (6–8). To the best of our knowledge, this is the largest study showing the association between arterial stiffness and LV diastolic function. Our results can provide more convincing evidence for the VVC concept based on the results obtained by analyzing a large number of subjects.

The association between increased arterial stiffness and diastolic dysfunction has been studied. In a previous large-scale study, cfPWV, the gold standard non-invasive method for estimating arterial stiffness, had a significant relationship with indicators of diastolic function (18). In another large observational study, cfPWV showed a significant association with e′ and E/e′, which are representative factors for evaluating of diastolic function after adjustment for cardiovascular risk factors (19). Also, arterial stiffness as measured by cfPWV tended to increase as the severity of diastolic dysfunction increased (8). Meanwhile, baPWV is more easily measurable than cfPWV and non-inferior to predict plaque progression and cardiovascular events (5, 20, 21). In a meta-analysis on 6,626 participants and 72 studies related to diastolic function, baPWV showed a higher correlation with diastolic parameters than cfPWV in patients with HFpEF (22). In a previous study using an invasive method, central pulse pressure and central systolic blood pressure were significantly correlated with E/e′ and e′ velocity; baPWV, as measured in all participants, showed similar results with central blood pressure in comparison to diastolic function (7). In another study of healthy subjects, decreased arterial stiffness measured by baPWV was associated with preserved LV diastolic function despite advanced age (23). The association between arterial stiffness and diastolic function was consistently observed in patients with hypertension, diabetes mellitus, and postmenopausal women (14, 24, 25). Our study results are in line with these previous findings. Compared to previous studies, the strength of our study is that it obtained robust results in a general population with non-specific disease patients with a larger sample size than other studies on VVC. Also, we demonstrated that baPWV shows relatively higher importance compared to other clinical variables affecting LV diastolic function such as obesity, blood pressure, or fasting glucose.

The mechanism by which increased baPWV is associated with diastolic parameter is unclear, but possible causes are as follows. If arterial stiffness is increased, the stiff arteries reflect the pressure pulse waves more rapidly toward the LV, which leads to an increase in pulse pressure (14, 26, 27). Increased systolic pressure causes LV hypertrophy and decreased diastolic pressure impairs coronary perfusion, which are thought to be a major cause of LV diastolic dysfunction (13, 28). While cfPWV measures only the stiffness of the central artery, baPWV measures the stiffness of both central and peripheral arteries, which may better explain the effect of afterload leading to diastolic dysfunction (29, 30). Shared common risk factors, such as aging, high blood pressure, hyperglycemia, dyslipidemia and chronic inflammation, may be possible underlying pathophysiologies linking increased arterial stiffness and LV diastolic dysfunction (5). Further studies are needed to confirm the detailed mechanisms and causal relationships between increased baPWV and diastolic dysfunction.

Left atrial (LA) enlargement is a structural marker for diastolic dysfunction (31), and it is closely related to diastolic dysfunction and cardiovascular prognosis (32). Based on this, LAVI has been used as one of the main indicators in the guideline for diastolic function (13). However, LA size might not be enlarged when mitral inflow is normal in the initial stage of abnormal myocardial relaxation (33). In addition, when the stroke volume is increased in, pregnant women, obese subjects or individuals with good physical conditioning, LA size could be increased, but show normal e′ velocity and myocardial relaxation pattern (33, 34). Our study result that LAVI was associated with baPWV in simple correlation analysis, but no significance after adjustment may have been attributed to various conditions affecting LA size besides diastolic dysfunction.

The strength of our study is to demonstrate the association between baPWV and echocardiographic parameters evaluating diastolic function in a large number of participants through various statistical analysis techniques such as univariable, multivariable logistic regression analysis, and random forest analysis. The GFR and hemoglobin, ranked high in the random forest analysis, are variables closely related to chronic kidney disease (CKD), which is known as one of the main pathophysiologies of HFpEF. The diagnosis and severity of CKD are determined based on GFR (35). In addition, in patients with CKD, erythropoietin production is decreased, and anemia is developed, which is one of the primary treatment goals for CKD patients (36). Renin-angiotensin system activation causes renal insufficiency and myocardial hypertrophy, which may be attributed to an increase of E/e′ and explain our result (37). Age, diabetes mellitus, and obesity are also well known as one the major causes of diastolic dysfunction, and fasting glucose ranks high in random forest analysis. The result that these variables are of high relative importance together with baPWV in our analysis results can be interpreted to demonstrate that baPWV is highly associated with echocardiographic variables that measure diastolic function.

Besides the retrospective design, our study has several limitations. First, causality between pharmacological treatment and abnormal diastolic function parameters could not be determined in this cross-sectional study. Also, a selection bias may be difficult to avoid because baPWV measurement was determined by the attending physicians. However, we attempted to overcome this limitation by enrolling a large number of participants. Secondly, we aimed to identify the association between baPWV and diastolic dysfunction. Still, many variables can affect both diastolic dysfunction and baPWV, so it is difficult to adjust all their interactions. Most participants underwent both echocardiography and baPWV on the same day. However, some participants had an interval of up to 7 days between two measurements. Thirdly, cfPWV is the gold standard for evaluating arterial stiffness non-invasively (38). However, baPWV is also a promising and non-inferior modality compared to cfPWV in previous studies (5, 20, 21, 29, 30). Fourthly, we did not perform an external validation of our random forest analysis model because of the single-center study. Further well-controlled multicenter prospective studies will be needed to validate the usefulness of baPWV for estimating VA coupling. Finally, since our study only enrolled the Korean population, caution should be exercised when applying the results to other ethnicities.

## Conclusions

In our large number of study subjects, arterial stiffness, as measured by baPWV, was correlated with LV diastolic indices. This provides stronger evidence for existing data about VVC. baPWV may be a clinically useful tool for measuring arterial stiffness as well as for identifying abnormal diastolic function.

## Data availability statement

The original contributions presented in the study are included in the article/Supplementary material, further inquiries can be directed to the corresponding authors.

## Ethics statement

The studies involving human participants were reviewed and approved by Institutional Review Board of Boramae Medical Center (Seoul, Republic of Korea). Written informed consent for participation was not required for this study in accordance with the national legislation and the institutional requirements.

## Author contributions

MK and H-LK performed the statistical analysis. MK drafted the manuscript. H-LK, W-HL, J-BS, S-HK, M-AK, and J-HZ reviewed/edited the manuscript and contributed to the interpretation of data. H-LK and J-HZ conceptualized the overall study design and supervised all aspects of the study and revised the manuscript. All authors contributed to the article and approved the submitted version.

## Conflict of interest

The authors declare that the research was conducted in the absence of any commercial or financial relationships that could be construed as a potential conflict of interest.

## Publisher's note

All claims expressed in this article are solely those of the authors and do not necessarily represent those of their affiliated organizations, or those of the publisher, the editors and the reviewers. Any product that may be evaluated in this article, or claim that may be made by its manufacturer, is not guaranteed or endorsed by the publisher.
