# Supplementary material for: Association between arterial stiffness and left ventricular diastolic function: A large population-based cross-sectional study
Source: Front Cardiovasc Med. 2022 Oct 13;9:1001248. doi: 10.3389/fcvm.2022.1001248 (PMC9606341; doi:10.3389/fcvm.2022.1001248)
Supplement: Supplementary file 1 [file Data_Sheet_1.docx]

**Supplementary Table 1. Binary logistic regression analysis showing the association between higher baPWV and abnormal diastolic parameters according to participants with healthy, having cardiovascular risk factors, and documented CAD.**

|  | Healthy (n=1,038) | | | CV risk factors only (n=4,102) | | | CAD (n=1,873) | |  |
| --- | --- | --- | --- | --- | --- | --- | --- | --- | --- |
|  | OR | 95% CI | *P*-value* | OR | 95% CI | *P*-value* | OR | 95% CI | *P*-value* |
| baPWV, m/s | 14.8 ± 3.1 | | | 16.1 ± 3.2 | | | 16.3 ± 3.4 | | <0.001† |
| Septal e' | 2.36 | 1.65-3.37 | <0.001 | 1.77 | 1.51-2.07 | <0.001 | 1.99 | 1.54-2.57 | <0.001 |
| Septal E/e' | 1.91 | 0.91-3.99 | 0.085 | 2.16 | 1.74-2.69 | <0.001 | 2.02 | 1.51-2.72 | <0.001 |
| LAVI | 1.15 | 0.72-1.82 | 0.566 | 1.20 | 1.01-1.42 | 0.034 | 1.04 | 0.82-1.31 | 0.766 |
| TR-Vmax | 2.80 | 1.14-6.87 | 0.025 | 2.70 | 1.98-3.69 | <0.001 | 2.43 | 1.68-3.50 | <0.001 |

* p-value for baPWV ≥16.1 m/s in septal e’ <7cm, baPWV ≥16.1 m/s in septal E/e’ ≥ 15, baPWV ≥16.3 m/s in LAVI ≥34 mL/m2, and baPWV ≥17.7 m/s in TR Vmax> 2.8 m/s, respectively.

† p-for-trend of baPWV according to the presence of risk factor or documented CAD

‡Four different multivariate analyzes were performed according to each dependent variable, and following clinical covariates were adjusted during the analysis: age, sex, hemoglobin, glomerular filtration rate. OR (95% CI) and P values are for in the association between baPWV and each diastolic parameter.

CV, cardiovascular; CAD, coronary artery disease; baPWV, brachial-ankle pulse wave velocity; OR, odds ratio; CI, confidence interval; LAVI, left atrial volume index; TR-Vmax, maximal velocity of tricuspid regurgitation;

**Supplementary Table 2. Variance inflation factor (VIF) values of independent variables for estimating diastolic parameters in multiple linear regression analysis. All diastolic parameters were continuous variables.**

|  | E/e' (septal) | Septal e' | LAVI | Peak velocity of TR |
| --- | --- | --- | --- | --- |
| Age | 1.75 | 1.75 | 1.74 | 1.76 |
| Sex | 1.26 | 1.26 | 1.22 | 1.24 |
| Body mass index | 1.10 | 1.10 | 1.10 | 1.10 |
| Systolic blood pressure | 1.63 | 1.64 | 1.64 | 1.67 |
| Heart rate | 1.13 | 1.14 | 1.15 | 1.13 |
| Hypertension | 1.26 | 1.26 | 1.26 | 1.28 |
| Diabetes mellitus | 1.44 | 1.44 | 1.47 | 1.41 |
| Smoking | 1.13 | 1.13 | 1.14 | 1.12 |
| Coronary artery disease | 1.02 | 1.02 | 1.02 | 1.03 |
| Calcium channel blockers | 1.25 | 1.25 | 1.27 | 1.25 |
| Beta-blockers | 1.19 | 1.18 | 1.17 | 1.18 |
| RAS blockers | 1.29 | 1.30 | 1.31 | 1.29 |
| Statin use | 1.44 | 1.44 | 1.43 | 1.46 |
| White blood cell | 1.10 | 1.10 | 1.08 | 1.08 |
| Hemoglobin | 1.37 | 1.37 | 1.32 | 1.36 |
| Fasting glucose | 1.32 | 1.32 | 1.33 | 1.30 |
| Estimated GFR | 1.20 | 1.20 | 1.19 | 1.17 |
| Low-density lipoprotein | 1.28 | 1.28 | 1.27 | 1.28 |
| Triglyceride | 1.21 | 1.21 | 1.22 | 1.19 |
| High-density lipoprotein | 1.24 | 1.24 | 1.25 | 1.22 |
| C-reactive protein | 1.11 | 1.11 | 1.08 | 1.12 |
| baPWV | 2.43 | 2.45 | 2.43 | 2.51 |

RAS, renin-angiotensin-aldosterone system; GFR, glomerular filtration rate; baPWV, brachial-ankle pulse wave velocity

**
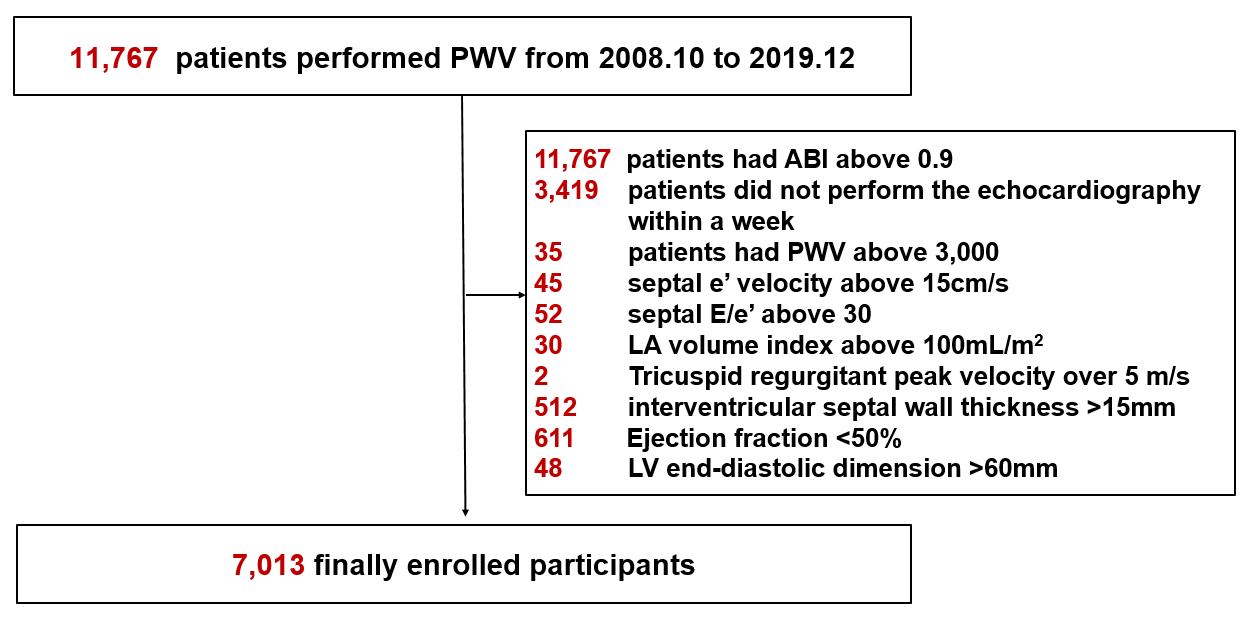
**

**Supplementary Figure 1. Study flow chart.** baPWV, brachial-ankle pulse wave velocity; ABI, ankle-brachial index; LA, left atrial; LV, left ventricular.


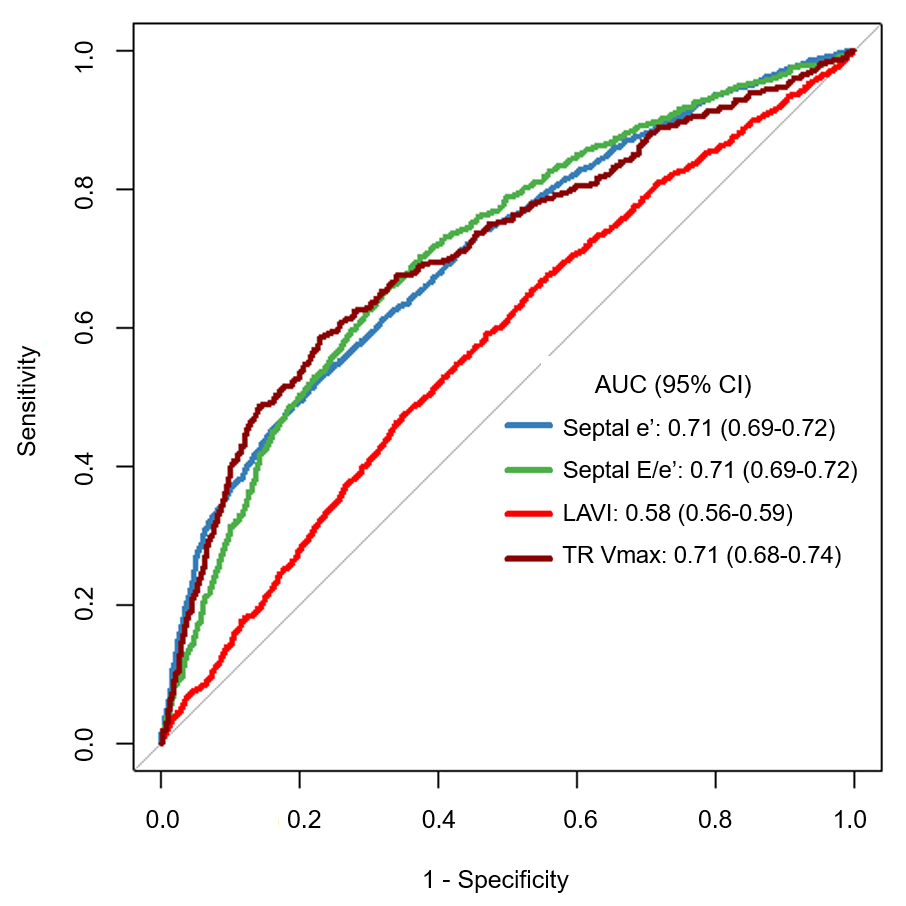


**Supplementary Figure 2. ROC curve for the baPWV to estimate abnormal diastolic function parameters.**
